# Uterine leiomyoma-like inflammatory myofibroblastic tumor: two case reports and literature review

**DOI:** 10.3389/fonc.2026.1801140

**Published:** 2026-04-22

**Authors:** Hongfei Liu, Guanghong Yang, Ruyu Zeng, Yuanli Zhong

**Affiliations:** 1 Department of Gastrointestinal Surgery, the Fourth Affiliated Hospital of School of Medicine, and International School of Medicine, International Institutes of Medicine, Zhejiang University, Yiwu, Zhejiang, China; 2 Department of Pathology, the Fourth Affiliated Hospital of School of Medicine, and International School of Medicine, International Institutes of Medicine, Zhejiang University, Yiwu, Zhejiang, China

**Keywords:** ALK, IMT, leiomyoma-like, pregnant, prognosis

## Abstract

Inflammatory myofibroblastic tumor (IMT) of the uterus is a rare low-grade malignant mesenchymal tumor characterized by its potential for recurrence and metastasis. The compact fibrous subtype can be easily misdiagnosed as a leiomyoma when it lacks a prominent inflammatory cell infiltrate. To enhance understanding of this disease and improve diagnostic vigilance, we present two cases of IMT exhibiting morphological features similar to leiomyomas for analysis. The ages of the two patients were 26 and 34 years, respectively. In Case 1, microscopic examination revealed densely packed spindle-shaped cells arranged in intersecting fascicles, with scattered inflammatory cells in the background and without myxoid stroma. The morphology closely resembled that of a leiomyoma, with only focal invasive growth. Case 2 involved a pregnant woman with a tumor that exhibited well-defined boundaries, varied cellularity, and compact regions with spindle cells arranged in bundles, along with stromal lymphocyte infiltration, while the loose areas demonstrated myxoid matrix. Immunohistochemical analysis indicated that both tumors were diffusely positive for ALK and expressed several myogenic markers. Fluorescence *in situ* hybridization (FISH) confirmed the presence of ALK gene breaks. A comprehensive understanding of these IMTs with leiomyoma-like features and accurate diagnosis are essential for effective patient treatment and prognosis.

## Introduction

Inflammatory myofibroblastic tumor (IMT) is a fibroblastic tumor characterized by low malignant potential, a recurrence rate of approximately 25%, and a metastasis rate of less than 2% (1, 2). This tumor is more prevalent in children and adolescents (3), with a higher incidence in females (3, 4). Although IMTs are commonly found in the pelvic and abdominal cavities, lungs, and bladder (5–7), primary involvement of the uterus is rare (8, 9). The histological morphology of IMT is diverse, typically presenting as a mixture of three growth patterns. When the tumor exhibits compact spindle cell morphology, immunohistochemical analysis often reveals myogenic markers, which can easily be confused with the more common uterine leiomyoma, potentially leading to misdiagnosis (10). Notably, patients with IMT are expected to derive significant benefits from targeted therapy using ALK kinase inhibitors (11). Given the specific biological behavior and potential therapeutic implications of IMT, accurate pathological diagnosis is essential. Here, we present two cases of uterine IMT that exhibit morphological features similar to leiomyoma, which are particularly susceptible to misdiagnosis as benign lesions, and discuss them in depth alongside a literature review. We aim to enhance the diagnostic vigilance of pathologists regarding this type of IMT and to prevent missed or incorrect diagnoses.

## Cases presentation

### Case 1

This case represents a consultation scenario within our hospital, initially diagnosed as a leiomyoma. The patient is a 26-year-old female who presented with a pelvic mass that had been discovered three years prior. An ultrasound examination revealed a hypoechoic mass measuring approximately 48 × 43 × 36 mm located on the anterior wall of the uterus. Consequently, a laparoscopic myomectomy was performed.

**Figure 1:**
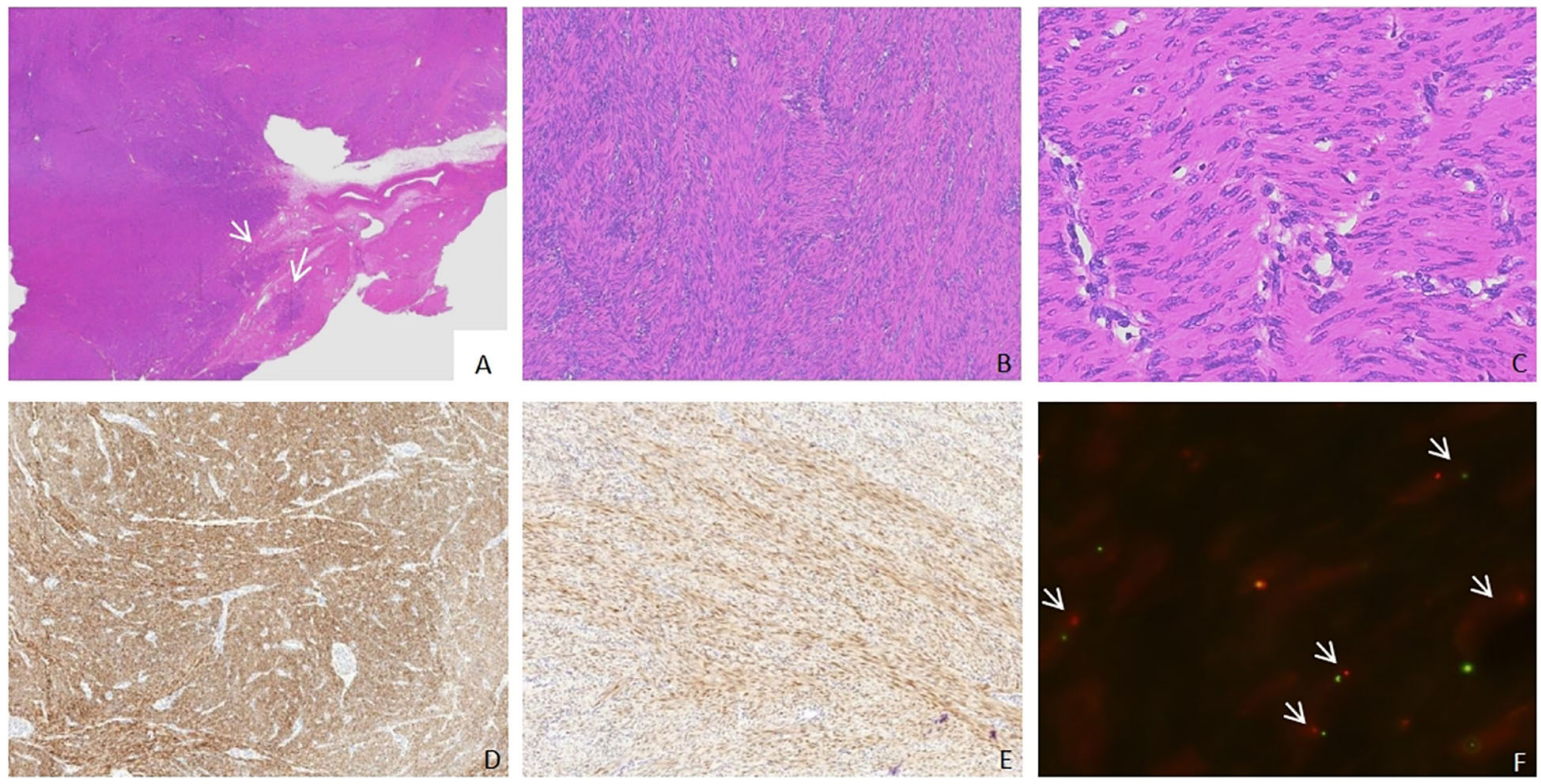
Case 1: pathological characteristics. **(A)** Displays the tumor's localized invasive margin (× 0.5), as illustrated by the arrow; **(B)** Reveals tumor cells arranged in bundles (× 4); **(C)** Illustrates tumor cells with a spindle shape, resembling smooth muscle cells, with a few inflammatory cells visible (× 20); **(D)** Desmin immunohistochemical staining demonstrates a widespread positive reaction in tumor cells (× 4); **(E)** ALK immunohistochemical staining also shows a widespread positive reaction in tumor cells (× 4); **(F)** FISH testing indicates the separation of ALK genes, as illustrated by the arrow (× 100).

The size and borders of the tumor could not be assessed due to fragmentation. The specimen had a gray-white, firm, whorled cut surface. Microscopically, the tumor boundary was indistinguishable, but focal infiltrative growth was observed (**Figure 1A**). The tumor cells were tightly arranged in bundles and fascicles (**Figure 1B**), displaying long spindle shapes with indistinct cell boundaries and rich fibrillar eosinophilic cytoplasm. The nuclei were elongated with rounded ends, fine chromatin, and no significant atypia, resembling smooth muscle cells morphologically (**Figure 1C**). Small or slit-like vessels were visible within the stroma, accompanied by scattered lymphocytic infiltration, while no significant myxoid matrix was noted. No necrosis or lymphovascular invasion was observed. Immunohistochemistry results demonstrated positivity for Desmin (**Figure 1D**) and estrogen receptor (ER), with intact FH expression, and a Ki-67 proliferation index of approximately 2%. A diagnosis of leiomyoma had been rendered at the original hospital. Additionally, our hospital conducted ALK testing, which yielded diffuse positivity (**Figure 1E**). Further ALK FISH break-apart probe detection indicated that approximately 40% of the tumor cells exhibited ALK gene breaks, interpreted as positive (**Figure 1F**). The final pathological diagnosis was a uterine IMT. Postoperative follow-up over a period of 10 months revealed no signs of recurrence or metastasis.

### Case 2

A 39-week pregnant patient was urgently admitted to the hospital due to vaginal discharge persisting for one hour, raising suspicion of premature rupture of membranes. Upon admission, a physical examination revealed grade II meconium staining. A subsequent vaginal examination and fetal heart rate monitoring indicated repeated decelerations, suggesting intrauterine distress and leading to the decision for an emergency cesarean section. During the surgery, after the successful delivery of the fetus, an exploratory assessment revealed a nodule approximately 10 mm in diameter beneath the endometrium, which the surgeon initially suspected to be a leiomyoma.

**Figure 2:**
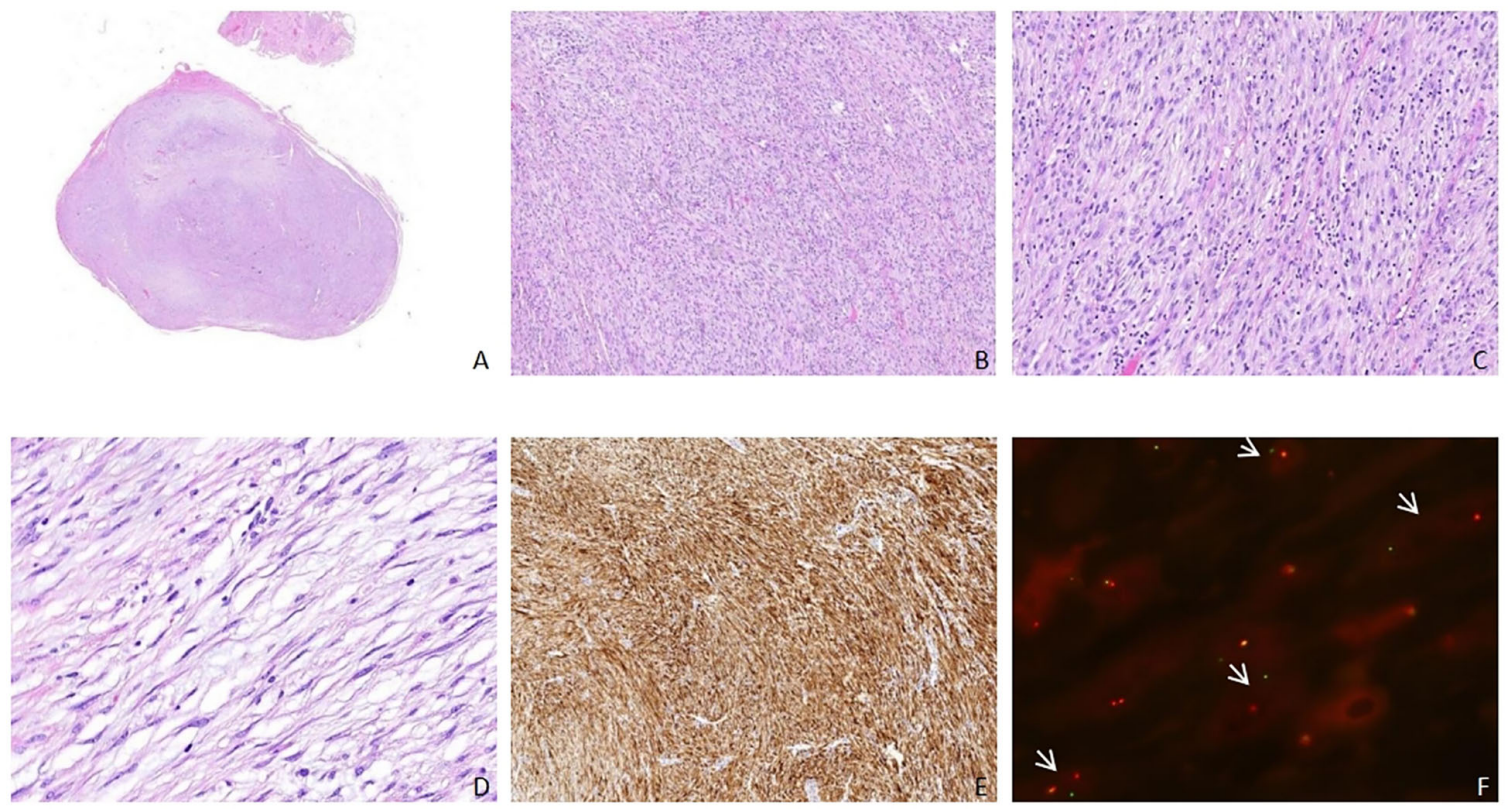
Case 2: pathological characteristics. **(A)** The tumor exhibits well-defined boundaries with a heterogeneous distribution of cell density (0.5); **(B)** In the tumor's denser regions, cells are abundant and organized into bundles (× 4); **(C)** The tumor cells are elongated and spindle-shaped, with notable lymphocyte infiltration present in the stromal tissue (× 10); **(D)** In the less dense areas, mucoid degeneration is evident (× 20); **(E)** ALK immunohistochemical staining reveals a widespread positive reaction in the tumor cells (× 4); **(F)** FISH testing demonstrates the separation of ALK genes, as indicated by the arrow (×100).

Pathological examination showed a uterine nodule measuring 11 × 7 × 5 mm, with a gray-white, firm cut surface. Microscopic examination revealed well-defined tumor boundaries (**Figure 2A**), showing varied cellularity, with certain regions exhibiting loose cell arrangements while others were densely packed. In the compact regions, the tumor cells appeared elongated and spindle-shaped, with abundant pale cytoplasm, fine chromatin, visible nucleoli, and were organized into bundles and fascicles, displaying mild atypia and occasional mitotic figures (**Figure 2B**). The stroma exhibited lymphocyte infiltration (**Figure 2C**), elongated thin-walled vessels, and focal collagen deposition. In the loose regions, the tumor cells were elongated and spindle-shaped, accompanied by myxoid matrix and inflammatory cell infiltration (**Figure 2D**). Immunohistochemical analysis revealed partial positivity for Desmin and positivity for CD10, while ER was negative and progesterone receptor (PR) was positive. P16 expression was partially positive, and P53 expression was consistent with wild-type. The Ki-67 proliferation index was approximately 5%. The initial pathological diagnosis was leiomyoma. Upon re-evaluation, additional ALK immunohistochemistry was performed based on the morphological features, revealing diffuse strong positivity in the tumor cells (**Figure 2E**). Further ALK FISH break-apart probe detection demonstrated that approximately 30% of the tumor cells exhibited ALK gene breaks, interpreted as positive (**Figure 2F**). The final pathological diagnosis was determined to be a uterine IMT. Postoperative follow-up over three months showed no signs of recurrence or metastasis.

## Discussion

IMT is a rare mesenchymal neoplasm characterized by a unique composition of myofibroblasts and fibroblast-like spindle cells, accompanied by inflammatory cell infiltration, as defined by the World Health Organization (WHO). This tumor is more prevalent in children and adolescents (3). Consistent with this, both patients presented in this report are young women. Notably, some literature suggests that IMT occurs more frequently during pregnancy than in non-pregnant women (12, 13). Pregnancy is a dynamic physiological process regulated by hormones, necessitating the remodeling and transformation of the endometrium to establish and maintain pregnancy. This unique physiological microenvironment may promote specific tumorigenic events in myofibroblasts, leading to the formation of IMT (14). Case 2 presented in this article exemplifies a pregnancy-related IMT. Clinically, uterine IMT often manifests as vaginal bleeding and pelvic masses. Pregnancy-associated IMT frequently exhibits distinct clinical features and is associated with complications such as gestational diabetes, preeclampsia, and premature rupture of membranes. Often, the tumor is identified or discovered at the time of delivery or during the postpartum period. The clinical manifestations of the two patients in this article align with existing literature reports.

Microscopically, IMT can exhibit either expansive or infiltrative borders. Its histological morphology primarily manifests in three patterns (15–17): 1. Myxoid type: the most common variant, resembling nodular fasciitis or granulation tissue. 2. Hypocellular fibrous type: characterized by a sparse arrangement of fibroblasts within hyalinized collagen fibers, accompanied by scattered inflammatory cell infiltration. 3. Spindle cell compact type: composed of tightly packed spindle cells arranged in bundles or a herringbone pattern. The tumor cells exhibit spindle-shaped nuclei, morphologically akin to smooth muscle cells, with stroma displaying variable amounts of myxoid matrix and infiltration by lymphocytes and plasma cells. Both cases reported in this article exhibit features characteristic of the cellular type. In Case 1, the tumor cells demonstrated the typical morphological characteristics of leiomyoma, specifically long spindle cells arranged in bundles, eosinophilic cytoplasm, and cigar-shaped nuclei, with no myxoid matrix in the stroma and few inflammatory cells. Crucially, the key microscopic distinction from leiomyoma lies in the focal infiltrative growth pattern of the tumor, which markedly differs from the well-defined borders of leiomyoma. In Case 2, although the dense spindle cell area resembles leiomyoma, the infiltration of inflammatory cells in the stroma and focal myxoid matrix suggest the possibility of IMT. Notably, IMT during pregnancy may also present with decidual-like changes. Furthermore, recent literature has documented a highly inflammatory cell-rich subtype that is frequently misdiagnosed as either a reactive lesion or an IgG4-related lesion (16).

The immunophenotype of IMTs overlaps with that of uterine leiomyomas and endometrial stromal tumors (18–20), often expressing markers such as desmin, smooth muscle actin (SMA), and CD10, which complicates differential diagnosis. Both cases reported in this article exhibited myogenic markers, with Case 2 additionally expressing CD10. ALK is a crucial marker for distinguishing these tumors, as approximately 90% of IMTs demonstrate positivity for ALK via immunohistochemistry (1, 17, 20), typically associated with ALK gene rearrangements. Both cases presented in this article showed diffuse strong positivity for ALK.

Molecular genetic studies indicate a strong association between uterine IMT and tyrosine kinase gene fusions, with ALK gene rearrangements being the most common, occurring in more than 80% of cases, which is significantly higher than the incidence in extrauterine IMT cases (21). Other kinase genes involved include RET, ROS1, and NTRK (22–25). Currently identified ALK fusion partners include IGFBP5, THBS1, FN1, DES, TIMP3, DCTN1, SEC31A, TPM3, and PPP1CB (25–28). Current research indicates that different fusion partners do not significantly impact the pathological morphology, prognosis, or treatment of IMT (10, 23). ALK gene rearrangement is the desirable criterion for diagnosing IMT. Both cases in this article were positive for ALK gene breaks by FISH. However, FISH detection has certain limitations: when the ALK fusion partner (such as IGFBP5 or NF1) is also located near ALK on chromosome 2, FISH detection may yield false-negative results; in such cases (27), ALK positivity by immunohistochemistry can serve as an important auxiliary diagnostic basis. Additionally, when IMT has ROS1, RET, or NTRK rearrangements, ALK FISH detection may also yield false-negative results (22–25). Therefore, when morphological features suggest IMT but ALK FISH is negative, next-generation sequencing (NGS) should be performed to confirm the diagnosis.

Existing evidence suggests that most IMTs exhibit a benign clinical course (13, 29); however, the recurrence rate remains approximately 25%, with a metastasis rate of less than 2% (1, 2). Ladwig et al. (30) proposed a risk assessment model assigning 1 point for each of the following criteria: age >45 years, tumor size ≥5 cm, mitotic count ≥4/10 high-power fields (HPF), and infiltrative borders. A score of 0 indicates no aggressive outcome, while a score of 1–2 points correlates with a 21% likelihood of aggressive outcomes; a score of ≥3 points indicates a definitive aggressive outcome. Based on this framework, Ladwig further introduced a “two-step classification model”: initially, clinical and pathological risk scores are applied to differentiate between low-risk and high-risk tumors, with recommendations for further molecular testing for intermediate-risk tumors. Studies have demonstrated that, in addition to ALK gene fusions, aggressive IMTs often present with other pathogenic gene alterations and extensive copy number variations, whereas benign IMTs are predominantly driven by ALK gene fusions alone. In the present report, Case 1 was classified as intermediate risk due to its infiltrative borders (1 point), indicating that further molecular testing was theoretically warranted to confirm the presence of additional pathogenic gene alterations. Unfortunately, this case was a consultation specimen and could not undergo additional testing. The patient has been followed for 10 months without recurrence or metastasis. Although previous literature has characterized leiomyoma-like IMTs as having a benign course without recurrence or metastasis, Devins et al. (10) first reported a case of IMT that exhibited a morphology indistinguishable from that of a leiomyoma and presented with abdominal metastasis, with a follow-up duration extending to 100 months. The primary tumor showed morphological characteristics consistent with a leiomyoma, while a second left paratubal soft tissue metastasis was observed 105 months later, displaying increased cellular atypia and mitotic activity compared to the initial presentation. Therefore, the biological behavior of leiomyoma-like IMTs may necessitate longer follow-up to clarify their prognosis. Case 2 falls within the low-risk group. In conjunction with existing literature, IMTs during pregnancy may resemble placental tissue, behaving as transient tumors that are often expelled during delivery and typically have a favorable prognosis (14). Furthermore, some studies suggest that lymphovascular invasion indicates a higher risk (30); however, neither case presented in this article demonstrated lymphovascular invasion. TP53 mutations and loss of p16 expression may correlate with the aggressive behavior of IMTs (31), yet immunohistochemical analysis of Case 2 revealed no abnormal expression of p53 or p16, reinforcing its benign nature.

Complete surgical resection is considered the preferred treatment for uterine IMT. For patients with aggressive IMT, the administration of tyrosine kinase inhibitors, such as crizotinib, may represent an effective alternative treatment strategy (11). Notably, a case reported by Yorita et al. (32) described a patient with multiple uterine leiomyomas who was treated orally with a gonadotropin-releasing hormone agonist (GnRH-a). This treatment resulted in a reduction in the largest lesion from 81 mm to 62 mm. Postoperative pathology confirmed that the largest lesion was an IMT, suggesting that uterine IMT may respond favorably to GnRH agonists.

Pickett’s systematic study of uterine mesenchymal tumors indicates that up to 0.3% of these tumors can be classified according to the current IMT diagnostic criteria (1). The primary differential diagnosis for uterine IMT is uterine leiomyoma. Given the rarity of reported IMT cases that exhibit morphological similarities to leiomyomas (18, 33), it seems impractical to routinely perform ALK immunohistochemistry on every case of leiomyoma. Consequently, identifying subtle morphological clues is particularly important, such as scattered inflammatory cell infiltration, focal myxoid degeneration, and infiltrative growth patterns. In this paper, Case 1 is morphologically very similar to a benign leiomyoma; however, it lacks significant inflammatory cell infiltration and a myxoid background, instead only displaying focal infiltrative boundaries, which may have important implications. In Case 2, the stroma shows mucoid changes and inflammatory cell infiltration, which are features of typical IMT morphology. The misdiagnosis of IMT as a smooth muscle tumor of uncertain malignant potential (STUMP) or myxoid leiomyosarcoma is not uncommon. Devereaux KA et al. (19) reviewed 43 cases of uterine and cervical STUMP, among which 6 cases (14%) tested positive for ALK immunohistochemistry. All 6 ALK-positive tumors exhibited myxoid features. Therefore, when STUMP presents with myxoid changes, conducting additional ALK immunohistochemistry and molecular testing is essential for establishing a definitive diagnosis. In Parra-Herran’s (34) research series on myxoid leiomyosarcoma, four out of thirty cases were misdiagnosed as myxoid leiomyosarcoma, whereas the actual diagnosis was IMT. Among these cases, three tumors tested positive for ALK. However, subsequent FISH testing on two of these cases returned negative results. The authors concluded that the ALK expression detected via immunohistochemical methods does not correlate with the typical rearrangement of the ALK gene. However, the absence of next-generation sequencing in the study to eliminate the possibility of ALK fusion with genes on the same chromosome—potentially resulting in false-negative ALK FISH results—suggests a cautious approach to classifying these cases as myxoid leiomyosarcoma. Notably, irrespective of the diagnostic classification criteria employed, the application of clinical and pathological risk stratification scores for uterine IMT typically categorizes these cases as high-risk. When using criteria for uterine smooth muscle tumors, they may be diagnosed as malignant. Importantly, if it is confirmed that these cases harbor targeted ALK rearrangements, patients could significantly benefit from targeted therapy. Furthermore, the secondary differential diagnosis for uterine IMT includes endometrial stromal tumor (EST). Particularly for high-grade endometrial stromal sarcomas exhibiting ZC3H7B-BCOR and BCOR internal tandem duplications (ITD) (35, 36), such tumors can also demonstrate spindle cell arrangements and myxoid changes in the stroma. The differentiation between these two entities primarily depends on ALK immunohistochemistry and FISH testing, alongside the characteristic molecular genetic alterations associated with endometrial stromal tumors.

## Conclusion

Uterine IMT is a rare mesenchymal tumor that poses significant challenges for clinical diagnosis. Pathologists must meticulously examine morphological features that resemble leiomyomas, searching for subtle indicators microscopically, such as inflammatory cell infiltration, stromal myxoid changes, and infiltrative margins. Given the higher incidence of IMT during pregnancy, heightened vigilance is required when assessing leiomyoma-like tumors exhibiting stromal inflammatory cell infiltration, particularly in pregnant patients. Immunohistochemical staining for ALK and FISH molecular testing are essential for accurate diagnosis. Furthermore, due to the poorly understood biological behavior of leiomyoma-like IMT, patients require extended follow-up to better assess their prognosis.

## Data Availability

The original contributions presented in the study are included in the article/supplementary material. Further inquiries can be directed to the corresponding author.
